# Predictors of early infection in cerebral ischemic stroke

**Published:** 2016

**Authors:** WMR Ashour, AD Al-Anwar, AE Kamel, MA Aidaros

**Affiliations:** * Department of Neurology, Faculty of Medicine, Zagazig University, Egypt

**Keywords:** ischaemic stroke, pneumonia, urinary tract infection, immunodepression, post-stroke infection

## Abstract

**Background:** Infection is the most common complication of stroke.

**Aim:** To determine the risk factors and predictors of post-stroke infection (PSI), which developed within 7 days from the onset of acute ischemic stroke.

**Subjects:** The study included 60 ischemic stroke patients admitted in the Neurology Department of Zagazig University, Egypt, who were subdivided into: [Non Stroke Associated Infection group (nSAI); 30 patients having stroke without any criteria of infection within 7 days from the onset and Stroke Associated Infection group (SAI); 30 patients having stroke with respiratory tract infection (RTI) or urinary tract infection within 7 days], in addition to 30 healthy sex and age-matching subjects as control.

**Methods:** All the patients had a detailed history taking, thorough clinical general and neurological examination, laboratory tests (Urine analysis & urine culture, blood sugar, lipid profile and serum tumor necrosis factor-alpha (TNF-α) and interleukin (IL)-10), a chest radiography to assess RTI and brain computed tomography (CT) to exclude the hemorrhagic stroke and to confirm the ischemic stroke.

**Results:** SAI patients were found to be significantly older with higher baseline blood glucose level. Also the number of patients with tube feeding, lower conscious level, more stroke severity and more large size infarcts were significantly higher in SAI patients. There was a significant elevation in the IL-10, a significant decrease in the TNF-α and a significant decrease in the TNF-α/ IL-10 ratio, in the SAI group. The baseline serum level of IL-10 ≥ 14.5 pg/ ml and size of infarct area > 3.5 cm3 were found to be the independent predictors of PSI.

**Conclusion:** Patients with older age, tube feeding, lower conscious level, worse baseline stroke severity, large cerebral infarcts in CT scan, and increased IL-10 serum level were more susceptible to infection. The baseline serum level of IL-10 ≥ 14.5 pg/ ml and the size of infarct area > 3.5 cm3 were the independent predictors of PSI.

## Introduction

Stroke remains a tremendous public health burden, as it is the leading cause of major long-term disability in adults and the third leading cause of death in developed countries [**1**].

While acute stroke patients may survive the initial brain insult, many succumb to subsequent complications over time. Infection is the most common of these complications and the chief cause of morbidity and mortality in the stroke surviving [**2**].

Previous studies showed a wide range of post-stroke infection rates, from 5%-65% for infections, 1%-33% for respiratory tract infection (RTI), and 2%-27% for urinary tract infection (UTI). A high percentage of these infections occur early in the first week after stroke [**3**].

In a meta-analysis [**4**], Westendorp et al. (2011) showed that infections commonly complicate the acute phase after stroke. The pooled overall infection rate was of 30% and the RTI and UTI occurred each in 10% of the patients.

Differences in patient populations, study design, and definition of infection may account for these large variations in post stroke infection rates [**5**].

Knowledge about the risk factors of post-stroke infections (RTI and UTI) are of obvious importance and will assist in the monitoring of patients and the prevention of stroke complications and could permit clinicians to provide the close surveillance and timely treatment of patients with stroke at a highest risk for infective complications, thereby optimizing the clinical outcomes [**6**].

Many studies examined risk factors and predictors for post-stroke infection, which included clinical, laboratory and radiological factors [**4**].

In addition, the acute stroke may lead to stroke-induced immunodepression, a systemic anti-inflammatory response that is related to susceptibility of infection. Certain features of this response were more pronounced in patients developing post stroke infections [**7**].

## Aim of work

This study was performed in acute ischemic stroke patients to achieve the following aim: determine the risk factors and predictors of post-stroke infection, which developed early in the acute ischemic stroke patients within the first week from the onset of stroke.

## Subjects

The study included 60 ischemic stroke patients admitted in Intensive Care Unit (ICU) and the Stroke Unit of the Neurology Department, in Zagazig University subdivided into: [Group I: non Stroke Associated Infection (nSAI) group - 30 patients having stroke without any criteria of infection within 7 days from the stroke onset and Group II: Stroke Associated Infection (SAI) group - 30 patients having stroke with Respiratory Tract Infection (RTI) or Urinary Tract Infection (UTI) within 7 days from the stroke onset], in addition to 30 healthy sex and age-matching subjects as control.

## Methods

A-Inclusion criteria:

Patients with first-ever hemispheric ischemic stroke within 24 hours from insult (stroke was diagnosed according to the World Health Organization (WHO) criteria [**8**] confirmed by brain Computed Tomography (CT). 

B-Exclusion criteria:

The following patients were excluded: 1-Hemorrhagic stroke. 2-Recurrent ischemic stroke. 3-Pathological brain atrophy. 4-Evident infection. 5-Evident inflammatory and/ or autoimmune disease. 6-Evident hematological disease. 7-Severe renal insufficiency. 8-Severe hepatic insufficiency. 9-Concurrent malignancy. 10-Surgical procedure within the past 12 months. 11-Certain drug intake (anti-inflammatory-immunosuppressive drugs). 12-Patients who died within the first 2 weeks after insult. 

-The exclusion criteria applied to stroke patients were also applied to the control group.

All the patients were assessed according to the following scheme of clinical evaluation and investigations:

1-Detailed medical history. 

2-Complete general examination. 

3-Complete Neurological examination: All the patients in our study were subjected to the following: a-Full neurological examination according to the items included in the examination sheet. b-Assessment of severity of stroke by using the National Institutes of Health Stroke Scale [**9**] (NIHSS).

- In our study, a special stress was done on the presence of risk factors according to criteria determined by Leoo et al. (2008) [**10**]:

a) Hypertension. b) Diabetes Mellitus. c) Hyperlipidemia. d) Cigarette smoking. e) Cardiac diseases.

**Investigations (for both the patients and controls):**

 A-Laboratory investigations:

-Complete Blood Count (CBC) including Total Leucocytes Count (TLC) (repeated as needed).

- Urine analysis & urine culture.

- Liver function tests.

- Renal function tests.

- Fasting and post-prandial blood sugar.

- Lipid profile.

- Serum uric acid.

- Erythrocyte sedimentation rate (ESR).

- Serum level of C-reactive protein (CRP). 

B- Special investigations:

-Blood samples were obtained to assess serum tumor necrosis factor-alpha (TNF-α) and interleukin-10 (IL-10 ) in 3 occasions:

* First occasion: On admission.

* Second occasion: On the 4th day.

* Third occasion: On the 7th day.

- Ratio between TNF-α and IL-10 was calculated as a measure of T helper (h) 1/ Th2 balance (proinflammatory and cellular immunity vs. anti-inflammatory and humoral immunity). 

C- Radiological investigations:

1- Chest plain X-ray performed to assess RTI (it was performed and repeated when indicated).

2- Brain Computed Tomography (CT) scans were performed within the first 24 hours to exclude hemorrhagic stroke and to confirm ischemic stroke.

3- Follow-up CT was performed after 48 hours.

-Upper respiratory tract infection defined as (2 or more of the following criteria: purulent sputum, fever > 38◦C; WBC >11.000 cell/ mL or < 4.000 cell/ mL, and normal chest X-ray films, no rales or rhonchi on auscultation and/ or dullness on percussion, no requirement of supplemental oxygen).

-Pneumonia (3 or more of the following: purulent sputum, fever > 38◦C; WBC > 11.000 cell/ mL or < 4.000 cell/ mL, new infiltrate on chest radiograph, requirement for supplemental oxygen) [**11**].

-Urinary tract infection defined as low urinary tract symptoms (such as frequent urination, dysuria, and flank pain) with a positive urine culture for an uropathogen (> 105 colony-forming units [cfu]/ mm3) or fever with a positive urine culture for an uropathogen in the absence of other infectious source [**12**].

- Non-septic fever was described as temperature 37.5°C without symptoms or signs of infection and Total Leucocytes Count (TLC) < 11.000 cell/ mL or > 4.000 cell/ mL [**13**].

- As the use of Foley catheters increases the risk of UTI after stroke, the limited use of catheterization may prevent UTI after stroke [**14**]. Therefore, in our study, the catheterization was done only if the patient was in a deep coma or had a urine retention or obstruction.

## Results

Results are shown in the following tables:

**Table 1:** Baseline characteristics of the study population

Baseline clinical and laboratory data	SAI (n = 30) (Mean ± SD)		nSAI (n = 30) (Mean ± SD)		Test of significance	P
**Age** (years)	72.0 ± 5.16		67.16 ± 4.89		**t** = 3.72	0.000**
Gender						
*Male*	17 (56.7%)		16 (53.3%)		**X2** = 0.06	0.79
*Female*	13 (43.3%)		14 (46.7%)			
**Systolic Blood Pressure** (mmHg)	158.16 ± 23.94		151.16 ± 21.2		**t** = 1.19	0.23
**Diastolic Blood Pressure ** (mmHg)	90.83 ± 10.99		89.5 ± 10.28		**t** = 0.48	0.62
**Blood Glucose Level ** (mg/ dl)	214.83 ±107.33		147.83 ± 80.51		**t** =2.73	0.008**
**Total Cholesterol** (mg/ dl)	203.66 ± 65.09		205.66 ± 65.32		**t** = 0.11	0.58
**Temperature ** (C°)	37.23 ± 0.89		37.1 ± 0.95		**t** = 0.55	0.58
	n	(%)	n	(%)	X2	
Tube feeding						
*Yes*	19	63.3	7	23.3	9.77	0.002**
*No*	11	36.7	23	76.7		
Catheter						
*Yes*	11	36.7	6	20	2.05	0.15
*No*	19	63.3	24	80		
Consciousness						
*Conscious*	13	43.4	25	83.3	10.3	0.001**
*Lower conscious level*	17	56.6	5	16.7		
Baseline NIHSS (severity)	17.06 ± 2.42		8.23 ± 2.83		12.97	0.000**
**CT findings ** (Side, Site & Size of infarct area)						
*Right side*	16	53.3	19	63.3	0.61	0.43
*Left side*	14	46.7	11	36.7		
Cortical	8	26.7	10	33.3	2.65	0.26
Cortico-subcortical	17	56.7	11	36.7		
Deep	5	16.7	9	30		
*size < 3 cm3*	3	10	20	66.7	20.4	0.000**
*size > 3 cm3*	27	90	10	33.33		
Serum levels of TNF-α, IL-10 & TLC						
*TNF-α (pg/ ml)*	14.39 ± 3.79		11.9 ± 3.05		3.12	0.002**
*IL-10 (pg/ ml)*	15.16 ± 3.46		12.7 ± 5.57		2.57	0.01*
*TLC (cell/ mL)*	7655.0 ± 1669.8		7160.0 ± 970.0		1.49	0.13
*Significant (P < 0.05) **Highly significant (P < 0.01)						

**Table 2:** Comparison between different patients groups regarding laboratory findings i.e. Serum levels of TNF-α, IL-10 and total leukocytes count TLC

Serum levels	SAI (n = 30) (Mean ± SD)		nSAI (n = 30) (Mean ± SD)		t	p
TNF-α (pg/ ml)						
*1st sample*	15.1 ± 3.05		13.67 ± 4.33		1.47	0.14
*2nd sample*	10.45 ± 3.85		13.88 ± 2.0		4.32	0.000**
*3rd sample*	12.73 ± 1.28		13.28 ± 0.97		1.91	0.06
IL-10 (pg/ ml)						
*1st sample*	16.29 ± 2.81		14.03 ± 3.71		2.66	0.01*
*2nd sample*	24.82 ± 8.58		17.02 ± 1.63		4.88	0.000**
*3rd sample*	15.96 ± 1.37		17.32 ± 0.74		4.76	0.000**
TLC (cell/ mL)						
*1st sample*	8153.33 ± 1739.94		7156.66 ± 1459.96		2.4	0.01*
*2nd sample*	11063.33 ± 2173.58		7703.33 ± 1305.55		7.25	0.000**
*3rd sample*	8800 ± 1232.04		7210 ± 727.46		6.08	0.000**
*Significant (P < 0.05) **Highly significant (P < 0.01)						

**Table 3:** Comparison between different patients groups regarding TNF-α/ IL-10 ratio

Sample	TNF-α/ IL-10 ratio		t	P
	SAI (Mean ± SD)	nSAI (Mean ± SD)		
1st sample	0.94 ± 0.23	1.07 ± 0.54	1.15	0.25
2nd sample	0.45 ± 0.17	0.93 ± 0.16	10.8	0.000**
3rd sample	0.8 ± 0.12	0.89 ± 0.06	3.75	0.000**
** Highly significant (P < 0.01)				

**Table 4:** Comparison between the occurrences of stroke associated RTI and UTI according to history of certain risk factors and certain clinical data

History & clinical data	RTI (n = 20)		UTI (n = 10)		X2	P
	n	(%)	n	(%)		
Hypertension	12	60%	4	40%	1.07	0.3
Diabetes	9	45%	3	30%	0.62	0.42
Hyperlipidemia	4	20%	6	60%	4.8	0.02*
Atrial fibrillation	4	20%	5	50%	2.85	0.09
Smoking	11	55%	0	0%	8.68	0.003**
Sex						
*Male*	14	70%	3	30%	3.28	0.07
*Female*	6	30%	7	70%		
Tube feeding						
*Yes*	14	70%	5	50%	1.14	0.28
*No*	6	30%	5	50%		
Catheterization						
*Yes*	5	25%	6	60%	3.51	0.06
*No*	15	75%	4	40%		
Consciousness						
*Conscious*	14	70%	3	30%	3.28	0.07
*Lower Conscious level*	6	30%	7	70%		
* Significant (P < 0.05) ** Highly significant (P < 0.01)						

**Table 5:** Forward stepwise logistic regression analysis of predictors of post-stroke infection i.e. independent predictors of infection

Models	OR (95% CI)	B	S.E	Sig.
Baseline IL-10 > 14.5 pg/ ml	6.01 (1.53-23.51)	1.79	0.69	0.01*
Infarct Size > 3.5 cm3	14.44 (3.22-64.77)	2.67	0.76	0.000**
*Significant (P < 0.05), Binary logistic regression was used to calculate P values and adjusted odds ratios (with 95% confidence intervals). B, Beta coefficient; S.E, Standard Error; CI, Confidence Interval; OR, Odds Ratio*				

## Discussion

Stroke is the main cause of disability and ranks second as a cause of death worldwide. The clinical course of stroke patients is not solely determined by the extent of brain damage and the resulting neurological deficit, but often complicated by post-stroke infections, with frequencies between 5 and 65% [**15**].

This study was designed to evaluate the post-stroke infection and the possible role of stroke-induced immunological mechanisms. Furthermore, the effect of infection upon stroke prognosis was assessed and an attempt to find out the most important predictors for that infection was made.

In the present study (**Table 1**), it was found that the SAI patients were significantly older than those without infection (72.5 ± 5.16 and 67.16 ± 4.89 years respectively). Our observation was similar to that obtained by Minnerup et al. (2010) [**16**] who demonstrated an advanced age as a risk factor for post-stroke infection. On the contrary, Hug et al. (2009) [**17**] reported no relation between age and the occurrence of post stroke infection.

It was found (**Table 1**) that patients with SAI had a significantly higher baseline blood glucose level than the patients without SAI (P = 0.008). In the same line with our results, Vermeij et al. (2009) [**18**], and Grabska et al. (2011) [**19**] demonstrated a significant relation between the blood glucose level and the occurrence of post stroke infection.

In our work (**Table 1**), the number of patients with tube feeding was significantly higher in patients with SAI than in those without infection (P = 0.002). This result goes hand in hand with that of Kwan & Hand (2007) [**20**] who reported that tube feeding in a stroke patient is the main predictor for subsequent infections.

The number of patients with a lower conscious level were significantly higher in the SAI group than in the nSAI group (P = 0.001) (**Table 1**) and this result is in agreement with Vermeij et al. (2009) [**18**].

Regarding the relationship between stroke severity (as measured by the National Institutes of Health Stroke Scale NIHSS on admission) and the occurrence of SAI, it was found that the NIHSS was significantly higher in patients with SAI (**Table 1**) i.e. patients with more severe strokes were at higher risk of post stroke infection. This was in agreement with Diedler et al. (2009) [**21**]. In contrast, Leira et al. (2009) [**22**] reported that stroke severity was not independently predictive for subsequent SAI.

In the present study (**Table 1**), it was observed that patients with larger strokes (> 3.0 cm3) were significantly more (P = 0.000) in the SAI group than in the non-SAI group (90% vs. 33.3% respectively), i.e. post-stroke infection increased with the increase of the size of the infarct area. The same was reported by Minnerup et al. (2010) [**16**] who found that the largest stroke size (> 5.0 cm3 or > 1/ 3 of the middle cerebral artery territory) was positively associated with RTI, UTI and other infections.

In this study (**Table 1**), the hemispheric lateralization (right or left) had no effect on the post-stroke infection (P = 0.43). These results are consistent with the results reported by Hug et al. (2009) [**17**] and Wartenberg et al. (2011) [**15**]. In contrast to these findings, Koch et al. (2006) [**23**] left-sided stroke was considered a risk factor for infectious diseases. Moreover, no significant differences could be found between the SAI group and the group without an infection regarding the site of infarction, cortical, cortico-subcortical or deep (P = 0.26) (**Table 1**). These results are consistent with the results of Hug et al. (2009) [**17**] and Minnerup et al. (2010) [**16**] in which a particular brain region predicting post-stroke infections could not be identified. In contrast, Walter et al. (2007) [**24**] reported a combined brainstem and cerebellar infarction as a risk factor for post-stroke infections.

In the current study (**Table 1**), a significantly increased baseline serum levels of IL-10 and TNF-α were found in stroke patients (both SAI & nSAI groups), compared to control individuals (P = 0.01 & 0.002, respectively). The same significant increase in the serum level of IL-10 in stroke patients were reported by Klehmet et al. (2009) [**7**]. In contrast, Kes et al. (2008) [**25**] observed significantly decreased levels of IL-10 in stroke patients, compared with the control. 

Nevertheless, Urra et al. (2009) [**26**] observed no significant difference in the baseline IL-10 levels. Concerning the TNF-α, a significantly increased baseline serum level of TNF-α was found in stroke patients, compared with the control. This result goes hand in hand with Haeusler et al. (2008) [**27**], who also demonstrated an elevated TNF-α in stroke patients. In contrast to our findings, Vogelgesang et al. (2010) [**28**] reported that stroke patients had a lower TNF-α level than the controls. However, Oto et al. (2008) [**29**] reported a different result, where they were not able to demonstrate any change of a TNF-α serum level in their stroke patients compared to the controls.

While the precise alterations in the human immune system induced by cerebral ischemia are still controversial, there is now a broad consensus that the ischemic stroke in humans results in a post-stroke immune suppression that predisposes to subsequent infection [**30**]. In our population, there were significant elevations of IL-10, a significant reduction of TNF-α, and a significant decrease of TNF-α/ IL-10 ratio in the patients with SAI than in the nSAI group. It was observed that the anti-inflammatory cytokine IL-10 serum level (**Table 2**) was significantly higher in patients with SAI than in patients without an infection (P = 0.000). In accordance with our study, Klehmet et al. (2009) [**7**] detected an early rise in plasma IL-10 in stroke patients who developed infections; they also mentioned that the infected patients tended to have higher IL-10 levels than the non-infected patients already from day one after stroke, which was most pronounced in patients who developed infections despite the preventive antibacterial therapy. A significant reduction of the level of the pro-inflammatory cytokine TNF-α (2nd sample) in patients with SAI than in those without infection (P = 0.000) was also observed in our study (**Table 2**). This was in accordance with Emsley and Hopkins (2008) [**3**], who reported a significant reduction of TNF-α in stroke patients with an infection relative to the non-infected. 

A significant decrease was found in TNF-α/ IL-10 ratio [which was used as a measure of T helper (h) 1/ Th2 balance (proinflammatory and cellular immunity vs. anti-inflammatory and humoral immunity)] (**Table 3**), compared to patients without infection in the second sample (P = 0,000). With these results, the existence of a stroke-mediated change in the immune system, i.e. stroke-induced immunodepression which had been reported in many other studies (Chamorro et al. 2007 [**31**], Haeusler et al. 2008 [**27**], Klehmet et al. 2009 [**7**], Urra et al. 2009 [**26**] and Vogelgesang et al. 2010) [**28**] could be confirmed, in which alterations in post stroke immune suppression include, but may not be limited to increased serum IL-10 concentrations, decreased serum TNF and decreased TNF-α/ IL-10 ratio. Thus, these findings suggested that SAI translates into an immunodepression state facilitated by the close interaction between the central nervous system and the immune system. 

Also it was found that the total leukocytes count (TLC) (**Table 2**) was significantly higher in patients with SAI than in those without infection (P = 0.000).

These results were consistent with those reported by Hug et al. (2009) [**17**] & Klehmet et al. (2009) [**7**]. 

Regarding the relationship between certain risk factors and the occurrence of post-stroke RTI or UTI (**Table 4**), it was found that patients with RTI had a significantly higher number with history of smoking compared with the patients with UTI. This result goes hand in hand with Sellars et al. (2007) [**32**]. Furthermore, it was found that the history of hyperlipidemia significantly associated with post-stroke UTI. Ovbiagele et al. (2006) [**33**] stated that patients who had atrial fibrillation and a history of hypertension were more likely to be diagnosed with a UTI. Matz et al. (2006) [**34**] stated that patients were more likely to experience post-stroke UTI if they had a history of diabetes.

The early identification of patients at high risk of post-stroke infection may promote and justify the intensive monitoring and tailored anti-infective treatment. After the adjustment of possible confounders in the logistic regression analyses (**Table 5**) in the current study, the serum level of IL-10 (> 14.5 pg/ ml) on admission and the size of the infarct area (> 3.5 cm3) were found as the earliest independent predictors of post-stroke infection. This result approximates that of Chamorro et al. (2006) [**35**] in which the baseline serum level of IL-10, the baseline monocytes count and the baseline NIHSS, were found as independent predictors of SAI. Hug et al (2009) [**17**] stated that the infarct size was the only independent early predictor for post-stroke infections. 

The same concerning infarct size was reported by Minnerup et al. (2010) [**16**], who stated that the largest stroke size (> 5.0 cm3) had a significant impact on post-stroke infection frequency and it was an independent risk factor for the occurrence of post-stroke infections.

## Conclusion

- Our findings support the notion that increased vulnerability of patients in the acute phase of stroke for infections can be attributed to either patients’ characteristics or/ and stroke-induced immunodepression. 

- Patients with older age, tube feeding, lower conscious level, worse clinical severity on admission, large cerebral infarcts on CT scan, and increased interleukin10 IL-10 serum level were more susceptible to infection.

- Knowledge of baseline factors that predict the occurrence of respiratory tract infection RTI and urinary tract infection UTI will assist in the monitoring of patients and the prevention of stroke complications. Our study identified the baseline serum level of IL-10 of more than 14.5 pg/ ml and a size of the infarct area of more than 3.5 cm3 as the independent predictors of post-stroke infection.

## Recommendations

- We recommend further studies to develop and validate the predictive tools (clinical scores, radiological findings and blood biomarkers) correspondent for the prediction of post-stroke infection; before the clinical manifestation.

- We also recommend a future research for the better understanding of the mechanisms underlying the brain-immune interaction after cerebrovascular stroke.
